# Age Differences among Female Sex Workers in the Philippines: Sexual Risk Negotiations and Perceived Manager Advice

**DOI:** 10.1155/2012/812635

**Published:** 2012-07-17

**Authors:** Lianne A. Urada, Robert M. Malow, Nina C. Santos, Donald E. Morisky

**Affiliations:** ^1^Division of Global Public Health, Department of Medicine, University of California San Diego, School of Medicine, 9500 Gilman Drive, MC 0507, La Jolla, CA 92093-0507, USA; ^2^Department of Health Promotion and Disease Prevention, Stempel College of Public Health and Social Work, Florida International University, 11200 SW 8th Street, AHC II-579A Miami, FL 33199, USA; ^3^School of Public Health, University of California, Berkeley. 50 University Hall #7360, Berkeley, CA 94720, USA; ^4^Doctoral Training Program in the Social and Behavioral Determinants of HIV/AIDS Prevention, Department of Community Health Sciences, 10833 Le Conte Ave, Fielding School of Public Health, University of California, CHS 26-070 Los Angeles, CA 90095-1772, USA

## Abstract

Consistent condom use among high risk groups such as female sex workers (FSWs) remains low. Adolescent female sex workers are especially at higher risk for HIV/STI infections. However, few published studies have compared the sexual risk negotiations among adolescent, emerging adult, and older age groups or the extent a manager's advice about condom use is associated with an FSW's age. Of 1,388 female bar/spa workers surveyed in the southern Philippines, 791 FSW who traded sex in the past 6 months were included in multivariable logistic regression models. The oldest FSWs (aged 36–48) compared to adolescent FSWs (aged 14–17) were 3.3 times more likely to negotiate condoms when clients refused condom use. However, adolescent FSWs received more advice from their managers to convince clients to use condoms or else to refuse sex, compared to older FSWs. Both adolescent and the oldest FSWs had elevated sexually transmitted infections (STIs) and inconsistent condom use compared to other groups. Having a condom rule at the establishment was positively associated with condom negotiation. Factors such as age, the advice managers give to their workers, and the influence of a condom use rule at the establishment need to be considered when delivering HIV/STI prevention interventions.

## 1. Introduction 

Approximately 40% of new HIV infections worldwide occur among those aged 15–24 years [1], with females representing approximately half of all infections [2]. Although HIV prevalence is low in the Philippines (under 1% of the general population), consistent condom use among high-risk groups such as female sex workers (FSWs) remains low as well (<30%) [3]. Therefore, FSWs working in night clubs/bars and spa/saunas remain at high risk of becoming exposed to HIV and other sexually transmitted infections (STIs) [4]. Approximately 40% of FSWs worldwide have entered the sex trade before the age of 18 [5]. Adolescent female sex workers are especially at higher risk for HIV/STI infections [6] because their genital tracts are not fully developed and consequently may tear more easily during penetrative sexual intercourse [7]. Studies have also revealed that FSWs who enter the sex industry as minors are more likely to experience sexual and physical abuse compared to FSWs who were not minors when they entered sex work [5]. However, few published studies have compared the sexual risk negotiations of older FSWs with younger FSWs, for example, adolescents (under 18 years old) and emerging adults (18–25 years old, an age marked by increased risk-taking and identity exploration) [8–10]. Furthermore, the extent a manager's advice is associated with age of an FSW is unknown for this region.

Research shows a greater occurrence of high-risk sexual behaviors among young FSWs compared to older FSWs. For example, two studies on FSWs from Thailand found that adolescent FSWs reported less condom negotiation and condom use and more anal sex compared to older FSWs [11, 12]. In Vietnam, a study showed how adolescent sex workers reported using condoms less frequently [13]. In addition to engaging in higher-risk behaviors on a more frequent basis, younger sex workers in South Asia had a greater number of sex clients per week and had less knowledge of HIV than their older counterparts [6, 7].

Other studies have shown how social and structural factors in the work environment affect FSWs' sexual risk behaviors [14–19]. In particular, combined government policy and community mobilization resulted in fewer HIV/STIs for venue-based FSWs in the Dominican Republic [17] and less unprotected sex in Brazil [20, 21]. For workplace policies and practices, a government sanctioned 100% condom use policy changed social norms and increased FSWs' access to STI treatment in Thailand [22, 23]. In the Philippines, combined manager and peer interventions in the workplace led to decreased HIV/STI risk among FSWs [24]. Also, the effects of sex work registration [25], the availability of condoms for venue-based versus street based FSWs [26], and the characteristics of FSWs working in higher risk venues in Mexico [27] and in China [28] have been studied. However, the impact of these workplace factors on younger compared to older FSW in entertainment venues is less known [29].

The primary aim of this paper was to examine how sexual risk negotiations differed among age groups of female sex workers (FSWs) in the southern Philippines, working in entertainment venues (e.g., night clubs, karaoke bars, spa/saunas) in the southern Philippines. This study hypothesized that younger aged FSWs (adolescents and emerging adult), compared to older FSWs, negotiated safer sex less. The study adjusted for the town in which they reside (controlling whether they received the intervention or not from a larger study), months worked as an entertainer, membership in an organization of workers, and whether a condom use rule existed at the establishment. The second aim of this paper was to determine the association between social and structural factors and an establishment manager's safer sex advice to an FSW. We hypothesized that older FSWs compared to younger FSWs received more positive safer sex advice from their managers. Findings from this study adds to the growing literature on the impact of social and structural influences on HIV/STI risk behavior by examining age differences among FSWs in addition to workplace factors.

## 2. Methods

The present study used cross-sectional follow-up data with 1,388 female entertainers involved in a longitudinal study in the southern Philippines. Only those entertainers who reported having paid sex in the previous 6 months (*N* = 791) were included in this analysis in order to restrict the sample to female sex workers. Participating city sites were originally randomized to intervention and comparison groups in the southern Philippines. To control for intervention effects, a city variable was included in this analysis.

The sample was originally assessed at baseline via interview-led surveys as detailed in Sirotin et al. (2006) [24] with 1,482 Filipinas working for establishments that required regular STI exams at government-run social hygiene clinics (SHCs). HIV tests were offered at these clinics. Although the establishments were mandated by the government to require their workers to attend the clinics, not all workers complied. Therefore, women were recruited from entertainment-related establishments (massage parlors, discos, bars, hotels, karaoke TV centers, and restaurants) on four southern Philippine islands, southern Luzon, Cebu, Ilo-Ilo, and northern Mindanao. Cities were randomized to 1 of 4 conditions, and establishment-based female workers were assigned to a peer intervention, manager intervention, combined peer-manager intervention, or no intervention group. Interviews were held in the SHC, business establishments, and residences. Approximately 98% of participants provided informed consent after receiving culturally sensitive human subjects' protection study information, approved by UCLA and the University of the Philippines Institutional Review Boards.

### 2.1. Measures

All measures included in this analysis were previously validated in the southern Philippines [30] during the formative stage of research and baseline survey data collection. The measures were extensively pretested, including the use of focus groups in the Philippines, to ensure that items were culturally and educationally appropriate according to the participants' literacy levels.

**Sociobehavioral Variables**

Age was recoded into four categories: 14–17, 18–25, 26–34, and 35–48 years old, based on the definition of emerging adulthood (18–25 years old), and categorized into ten-year intervals. The following variables were also collected: educational level, marital status, number of months worked as an entertainer, and the number of self-reported STIs during the previous six months. Injecting drug use experience (ever) was recorded as “Yes-No.” Consistent condom use was measured by whether the FSW “used condoms every time for vaginal sex,” with responses dichotomized as Yes (always) and No (not always).

**Sexual Risk Negotiation**

FSWs responded to “If a client refuses to use a condom, what do you usually do?,” with five choices of response categories: “still have sex with the client,” “refuse to have sex,” “force clients to use condoms,” “and explain condoms to clients,” “take medication/treatment after sex.” Although these categories may not seem mutually exclusive, the FSWs were instructed to choose one item. The items were first created from focus group data conducted with this population. For this analysis, categories were collapsed into (0) “no negotiation” if the FSW “still had sex with the client,” “took medication/treatment after sex,” or responded with “don't know,” and (1) “negotiation” if the FSW “refused to have sex,” “forced clients to use condoms,” and “explained condoms to clients.”

**Social-Structural Variables**

“What has your manager told you to do if your client refuses to use a condom?” had response choices: “manager never talks about condoms,” “have sex without a condom,” “refuse to have sex,” and “convince client to use a condom.” The response was recoded as “0” if they said the manager “never talks about condoms,” “says to have sex with client anyway,” or if the FSW said “don't know.” The response was recoded as “1” if the manager said “to refuse to have sex” or “to convince clients to use condoms.”

Other social-structural variables included the following measures which had dichotomized “Yes-No” answer choices: “Managers talked to you about using condoms,” “Does the establishment you work in have a rule that all workers must use condoms when having sex with clients?” and “You belonged to an organization of workers.”

### 2.2. Analyses

Bivariate and multivariable logistic regression were performed to identify the associations between age and sexual risk negotiation, and age and manager response to “if the client refuses to use condoms,” adjusting for potential confounders such as intervention effects (city), months worked, belonging to an organization of workers, and existence of a condom rule at the establishment. Models were developed using a manual procedure where all variables that attained a significance level <10% in bivariate models were considered in multivariate analyses in order of most to least significant. Variables that were collinear were inserted in the regression model one at a time, using a forward stepwise approach, and only retained if they remained statistically significant at the *P* < .05 level or if they were deemed important to retain to control for the abovementioned effects (months worked, city of interview, belonging to an organization of workers, and condom rule at the establishment).

## 3. Results

### 3.1. Sociobehavioral and Social-Structural Characteristics

Table 1 displays sociobehavioral and sociostructural characteristics by age category of the FSWs (*n* = 791) who participated in this study in the southern Philippines. In terms of sociobehavioral characteristics, adolescent FSWs (age 14–17-year-old) worked a median of 5 months, 11% were married or living with a boyfriend, their median number of children was 0, and they had a median of 8 years of education. Also, 9% reported ever using drugs, 32% having an STI in the past 6 months, 59% ever having an STI test, and 38% always using a condom when having vaginal sex. For sociostructural characteristics, 77% said a coworker tried to convince them to use condoms, 4% said they belonged to an organization of workers, 66% said a manager talked to them about using condoms, and 73% said they worked at an establishment with a rule that workers must use condoms with clients.

FSWs in the 18–25-year-old (emerging adulthood) age category worked a median of 8 months, 29% were married or living with a boyfriend, their median number of children was 1, and they had a median of 9 years of education. Also, 5% reported ever using drugs by injection, 25% having an STI in the past 6 months, 64% ever having an STI test, and 50% always using a condom when having vaginal sex. For sociostructural characteristics, 70% said a coworker tried to convince them to use condoms, 3% said they belonged to an organization of workers, 67% said a manager talked to them about using condoms, and 77% said they worked at an establishment with a rule that workers must use condoms with clients.

FSWs, 26–34 years old, worked a median of 15 months, 47% were married or living with a boyfriend, their median number of children was 1, and they had a median of 9 years of education. Also, 8% reported ever using drugs by injection, 29% having an STI in the past 6 months, 88% ever having an STI test, and 45% always using a condom when having vaginal sex. For sociostructural characteristics, 71% said a coworker tried to convince them to use condoms, 4% said they belonged to an organization of workers, 59% said a manager talked to them about using condoms, and 67% said they worked at an establishment with a rule that workers must use condoms with clients.

FSWs, 35–48 years old, worked a median of 36 months, 48% were married or living with a boyfriend, their median number of children was 2, and they had a median of 8 years of education. Also, 2% reported ever using drugs by injection, 43% having an STI in the past 6 months, 95% ever having an STI test, and 30% always using a condom when having vaginal sex. For socio-structural characteristics, 82% said a co-worker tried to convince them to use condoms, 7% said they belonged to an organization of workers, 72% said a manager talked to them about using condoms, and 83% said they worked at an establishment with a rule that workers must use condoms with condoms.

### 3.2. Associations between FSWs' Age and Sexual Risk Negotiation If a Client Refuses to Use Condoms

In the final multivariate model (Table 2), the 35–48-year-old age category was independently associated with sexual risk negotiation; this group was over 3 times more likely to negotiate condoms when a client refused to use condoms (AOR  =  3.33, 95% CI  =  1.27–8.71), compared to FSWs who were 14–17 years old. Significant covariates included the city where the interview took place (AOR  =  1.06, 95% CI  =  1.02–1.11) and the existence of a condom rule at the establishment (AOR  =  2.50, 95% CI  =  1.70–3.68).

### 3.3. Associations Between FSWs' Age and Perceived Manager Safer Sex Advice If a Client Refuses to Use Condoms

In Table 3, the 14–17-year-old age category was significantly associated with a manager's safer sex advice compared to the older age categories aged 18–25 (AOR = 0.17, CI = 0.64-0.46), aged 26–34 (AOR = 0.06, CI = 0.01–0.31), and aged 35–48 (AOR = 0.05, CI = 0.00–0.78) which were inversely associated with a manager's safer sex advice. No other covariates were significantly associated at the *P* < .05 level with a managers' safer sex advice in this multivariate model.

## 4. Discussion

In this study of female sex workers in the southern Philippines, nearly 1 in 4 reported having an STI in the past 6 months and nearly half said they used condoms consistently. However, those in the adolescent category (aged 14–17) and the oldest age group (aged 35–48) reported having more STIs and less consistent condom use than the 18–25 and 26–47-year-old age categories. Adolescent FSWs were less likely to negotiate condoms, compared to older FSWs. However, adolescent FSWs received more reinforcement from their managers to engage in safer sex compared to the older age groups. Findings from this study reinforce the need for interventions to target young female sex workers at risk for HIV/STI, as described in the literature due to the heightened susceptibility of younger FSWs to infections and violence [5]. However, this study's findings also illustrate the need to target the oldest FSW category (aged 35–48), a group less described in the existing literature.

As hypothesized, the oldest FSWs were three times more likely to negotiate condoms than the adolescent sex workers in this study. This finding may be related to other studies that describe the violence that might affect a younger FSW's ability to negotiate safer sex with unwilling clients. For example, in a cross-sectional study in Karnataka, India, the greatest percentage of FSW beaten or raped in the past year were among those under the age of 29 [31]. A sample of FSWs in Thailand also reported how 25% of workers under age 18 experienced sexual and physical violence in their work during the past week [12]. Further studies on the association between condom negotiation and violence, particularly with respect to age differences, may be needed for this population in the Philippines.

Unexpectedly, the oldest FSWs (aged 35–48) had a higher likelihood of a manager telling them “to do nothing” or “to have sex anyway” when a client refuses condoms. In addition, this study found that the oldest category of FSWs had the highest percentage of self-reported STIs in the past six months and the lowest consistent condom use, compared to other age categories. A study conducted in Andhra Pradesh, India, found older FSWs engaged in risky anal sex more often than younger FSWs due to the extra pressure to give into client's demands because of the FSW's advanced age [32].

FSWs who worked at establishments with a condom rule were 2.5 times more likely to negotiate safer sex with clients who refused to use condoms. Growing evidence shows that condom use and condom negotiation among female establishment-based sex workers are influenced by social and structural influences [15–19] and specifically that peer and structural interventions (i.e., having a 100% condom use rule) are effective [17, 22, 23].

The city in which the interview took place was also a significant covariate for condom negotiation in this population, presumably due to intervention effects. The effects of peer and manager interventions on younger versus older FSWs need further exploration and can be a topic of another study. For example, FSWs have organized into collective bargaining units that negotiated with police, brokers, and brothel owner for better STI treatment and condom use in India [33, 34]. Belonging to a workers' cooperative needs further investigation because it was not a significant factor in the present study, possibly due to the small number who belonged to an organization. Other studies have found that community mobilization among FSWs was a protective factor against HIV/STIs [35–37]. Therefore, community mobilization needs further exploration as well.

This study has several limitations. First, our reliance on self-reported survey data may have led to an underreporting of high-risk behaviors as well as other possible influences on the participants' responses. However, Morisky et al. (2006) assessed the reliability of a self-reported condom use scale and validated the findings with clinical STI data [38]. Second, the study's cross-sectional design makes inferences to causation impossible. Third, insufficient data existed for different types of partner or client relationships, such as more intimate regular partner relationships among the FSWs that could create the most health vulnerability [39, 40]. Age differences and partner characteristics could be the topic of another study.

## 5. Conclusions

This study's findings suggest that adolescent female sex workers (aged 14–17) in the Philippines negotiated safer sex less with clients who refused to use condoms, compared to older sex workers, in particular those in the highest age bracket (aged 35–48). However, this oldest group of sex workers experienced less positive safer sex advice given by their managers. Also, both adolescent and 35–48-year-old FSWs engaged in the highest levels of risky behavior in terms of STIs and inconsistent condom use. A presence of a condom rule at the current establishment was also a significant covariate for condom negotiation. Findings from this study imply that interventions need to occur at organizational and structural levels [26, 32] to address safer sex negotiation among the FSWs, taking into consideration factors such as age, the advice managers give to their workers, and the influence of a condom use rule at the establishment. More studies are needed that compare whether the youngest and oldest FSWs experience violence differently, especially for those who entered the sex trade under 18 and were forced/coerced into sex work acts. Comparisons between the emerging adulthood and older age categories for FSWs may particularly warrant further investigation because of the heightened risks normally associated with emerging adulthood.

## Figures and Tables

**Table 1:** Sociobehavioral and social-structural characteristics of female sex workers in the southern Philippines by age category (*N* = 791).

	Age category %				
	Total *N*	14–17	18–25	26–34	35–48
	(*n* = 791)	(*n* = 56)	(*n* = 496)	(*n* = 179)	(*n* = 60)
Sociobehavioral					
Months worked as an entertainer^∗^	10 (4–21)	5 (3–12)	8 (3–12)	15 (7–36)	36 (20–96)
Married or living with a boyfriend	264 (33)	6 (11)	144 (29)	85 (47)	29 (48)
Education^∗^	9 (7–10)	8 (7–9)	9 (7–10)	9 (7–10)	8 (6–10)
Number of children^∗^	1 (0–2)	0 (0)	1 (0-1)	1 (1–3)	2 (1.5–4)
Ever used drugs by injection	45 (6)	5 (9)	25 (5)	14 (8)	1 (2)
Any STIs during past six months	219 (28)	18 (32)	123 (25)	52 (29)	26 (43)
Ever had an HIV test	566 (72)	33 (59)	319 (64)	157 (88)	57 (95)
Always uses a condom when having vaginal sex	367 (46)	21 (38)	248 (50)	80 (45)	18 (30)
Social-structural					
Coworker ever tried to convince you to use condoms with clients	568 (72)	43 (77)	349 (70)	127 (71)	49 (82)
Belongs to an organization of workers	30 (4)	2 (4)	16 (3)	8 (4)	4 (7)
Your manager ever talked to you about using condoms	519 (66)	37 (66)	334 (67)	105 (59)	43 (72)
The current establishment has a rule that workers must use condoms	594 (75)	41 (73)	383 (77)	120 (67)	50 (83)

^∗^Median, IQR.

**Table 2:** Associations between female sex workers' age and sexual risk negotiation if a client refuses to use condoms in the Philippines (*N* = 791)^∗^.

Variable	Unadjusted odds ratio (95% confidence interval)	Adjusted odds ratio (95% confidence interval)
Age (versus 14–17)		
18–25	1.78 (1.01–3.16)	1.77 (0.97–3.22)
26–34	1.13 (0.61–2.10)	1.42 (0.73–2.74)
35–48	2.59 (1.13–5.93)^∗∗^	3.33 (1.27–8.71)^∗∗^
Months worked as an entertainer	1.00 (0.99-1.00)	1.00 (0.99-1.00)
City of interview^∗^	1.11 (1.07–1.16)^∗∗^	1.06 (1.02–1.11)^∗∗^
Sociostructural		
Belongs to an organization of workers	2.11 (0.80–5.59)	2.10 (0.76–5.80)
Manager talked about using condoms	2.29 (1.67–3.15)^∗∗^	1.37 (0.95–1.98)
Condom rule exists at establishment	3.43 (2.44–4.82)^∗∗^	2.50 (1.70–3.68)^∗∗^

*Controlling for intervention effects, **significant at  *P* < .05.

**Table 3:** Associations between FSW age and perceived manager safer sex advice if a client refuses condoms in the southern Philippines (*N* = 791)^∗^.

Variable	Unadjusted odds ratio (95% confidence interval)	Adjusted odds ratio (95% confidence interval)
Age (versus 14–17)		
18–25	0.17 (0.07–0.46)^∗∗^	0.17 (0.64–0.46)^∗∗^
26–34	0.08 (0.01–0.39)^∗∗^	0.06 (0.01–0.31)^∗∗^
35–48	0.12 (0.01–1.00)	0.05 (0.00–0.78)^∗∗^
Months worked as an entertainer	1.00 (0.99–1.02)	1.01 (0.99–1.03)
City of interview^∗^	0.92 (0.82–1.04)	0.91 (0.80–1.03)
Sociostructural		
Belongs to an organization of workers	2.65 (0.59–11.88)	3.01 (0.62–14.67)
Condom rule exists at establishment	0.88 (0.34–2.28)	1.05 (0.38–2.90)

*Controlling for intervention effects, **significant at  *P* < .05.
